# Ticks (*Acari: Ixodidae*) infestation on cattle in various regions in Indonesia

**DOI:** 10.14202/vetworld.2019.1755-1759

**Published:** 2019-11-11

**Authors:** Ana Sahara, Yudhi Ratna Nugraheni, Gautam Patra, Joko Prastowo, Dwi Priyowidodo

**Affiliations:** 1 Department of Parasitology, Faculty of Veterinary Medicine, Universitas Gadjah Mada, Yogyakarta, Indonesia; 2 Department of Veterinary Parasitology, College of Veterinary Science and Animal Husbandry, Aizawl, Mizoram, India

**Keywords:** cattle, *Haemaphysalis*, *Ixodidae*, *Rhipicephalus*, ticks

## Abstract

**Background and Aim:**

Ticks (*Ixodidae*) not only cause blood loss in cattle but also serve as vectors for various diseases, thus causing direct and indirect losses. Moreover, tick infestation can cause significant economic losses. This study aimed to identify the diverse species of ticks infesting cattle in five different regions in Indonesia.

**Materials and Methods:**

Tick specimens were obtained from local cattle in five different areas in Indonesia. The morphology of the specimens was macroscopically and microscopically evaluated, and the resulting data were descriptively and qualitatively analyzed.

**Results:**

In total, 1575 ticks were successfully collected from 26 animals. In total, two genera and three species, namely, *Rhipicephalus microplus*, *Haemaphysalis bispinosa*, and *Rhipicephalus pilans*, were identified. The cattle in Yogyakarta and Riau were infested by *H. bispinosa*, while the cattle in Sukabumi, Bali, and Lombok were infested by *R. microplus* and *R. pilans*. The level of infestation varied among regions, with *R. microplus* being the most commonly found species.

**Conclusion:**

The results of this study revealed that cattle in different regions of Indonesia were infested by variable numbers of tick species. In particular, the cattle in Yogyakarta and Riau were solely infested by *H. bispinosa*; this is a new finding in terms of the distribution of tick species in the country. Increased tick infestation in cattle decreases productivity and causes health problems; therefore, it deserves serious attention. Our findings can help in the formulation of an effective strategy for controlling and preventing cattle tick infestation in the country.

## Introduction

Ticks (*Ixodidae*) are ectoparasites that are commonly found in cattle. Direct effects of tick infestation in cattle include blood loss and weight loss, while indirect effects are often associated with the role of ticks as vectors of pathogenic diseases. Ticks are important vectors of various diseases that infect cattle, humans, and other vertebrates; almost 10% of 900 known tick species can spread pathogenic microorganisms to animals and humans [1]. Piroplasmosis and rickettsiosis are some of these diseases, and they adversely affect the health of cattle. Difficulties in raising cattle and health issues represent the main problems that affect cattle productivity in developing countries such as Indonesia [2,3]. It is expected that 80% of the worldwide cattle population, particularly those in tropical and subtropical countries, are directly and indirectly affected by tick infestation [4].

Statistical data from the Ministry of Agriculture in the Republic of Indonesia showed that, in 2017, the overall cattle population was approximately 16.6%, with 43% in Java, 25% in Timor, and 32% in various other islands. The distribution of ticks and the diseases spread by them is influenced by the climate. Ticks are widely distributed in tropical and subtropical regions. Tick infestation also results in significant economic losses in the cattle industry. Piroplasmosis (babesiosis and theileriosis) and rickettsiosis (anaplasmosis) are common protozoan diseases spread by ticks among cattle, and they have become endemic in various countries, causing several health problems in domestic ruminants [5,6]. South Asia houses several tick species; in fact, 97 species have been reported in this region [7]. However, the correlation between the resulting diseases and socioeconomic effects has not been established [8,9]. Some in-field studies show that tick species are often found on livestock, particularly cattle. Thus, further studies should investigate their abundance and identify the species. The majority of surveys and studies have evaluated *Rhipicephalus microplus* found on cattle. However, to the best of our knowledge, no study has performed a comprehensive mapping of tick species that infest cattle across Indonesia.

This study aimed to identify the species of ticks infesting cattle in five regions of Indonesia and determine the relative abundance in each region to provide information that can help in controlling and preventing cattle tick infestation in the country.

## Materials and Methods

### Ethical approval

The study was based on sampling and ethical approval was not necessary.

### Specimen collection

**Figure-1:**
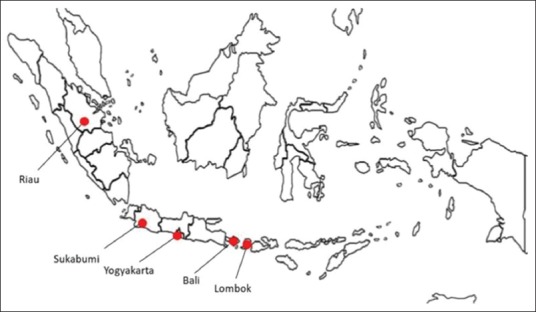
Geographical distribution of the sampling locations. Tick samples were collected on cattle from five locations across the Indonesian archipelago [Source: 10].

Tick specimens were collected from 26 local cattle in five different provinces in Indonesia: Yogyakarta, Riau, Sukabumi, Bali, and Lombok (Figure-1 [10] and Table-1). Certain parts of the body, including the head, back, tail, and legs, were carefully observed, and each tick was carefully picked from the surface using tweezers or a pair of thumb forceps. The collected samples were stored in 70% alcohol solution for identification.

**Table-1:** Distribution of every species collected from cattle in various areas.

Locations	Tick species	Number of cattle	Number of ticks	Body part
Yogyakarta	*H. bispinosa*	3	421 (29 males, nymph 28)	Ear, face, eyeless, udder, flank, and perineum
Sukabumi	*R. microplus*	6	806 (73 males, 28 nymph)	Belly, udder, external genitalia, flank, and perineum
	*R. pilans*		12 (3 male)	
Denpasar	*R. microplus*	3	19 (2 males)	Belly, abdomen, flank, udder, and perineum
Lombok	*R. microplus*	4	88 (12 males)	Belly, flank, udder, and perineum
	*R. pilans*		3 (1 male)	
Riau	*R. microplus*	10	226 (23 males, 17 nymph)	Ears, belly, abdomen, udder, and perineum

*R. microplus*: *Rhipicephalus microplus*, *R. pilans*: *Rhipicephalus pilans*, *H. bispinosa*: *Haemaphysalis bispinosa*

### Morphological identification

The collected tick specimens were counted and identified macroscopically and microscopically. For macroscopic evaluation, the specimens were washed with an ultrasonic device for approximately 10 min to separate them from the debris of their hosts. The washed specimens were then observed under a stereomicroscope (Olympus-CH20BIM). Identified ticks were microscopically evaluated after the preparation of glass slides. The specimens were kept in 10% KOH solution for 24 h, washed with an ultrasonic device, and gradually dehydrated in 70%, 80%, and 95% alcohol for 20 min each. Subsequently, the specimens were thinned by placement in a 95% alcohol and clove oil mixture for 7 min. The thinned specimens were glued on the glass slide using Canada balsam and kept in an incubator at 60°C for 7 days, following which they were observed under a stereomicroscope (Olympus-CH20BIM). The species were identified by referring to the identification keys proposed by Anastos [8] and Tanskull and Inlao [11].

Macroscopic evaluations primarily involved observations of the tick color and identification of any cavities or protrusions on their body surface. Microscopic evaluations were aimed at confirmation of the macroscopic findings, particularly in certain areas that could be observed only on the prepared slides.

## Results

In total, 1575 ticks were collected. Macroscopic and microscopic observations revealed two genera and three species: *R. microplus*, *Rhipicephalus pilans*, and *Haemaphysalis bispinosa*. The proportion of each species and their distribution in each area are summarized in Tables-1 and 2. The cattle in Yogyakarta and Riau were infested by *H. bispinosa*, while the cattle in Sukabumi, Bali, and Lombok were infested by *R. microplus* and *R. pilans*. The level of infestation varied among regions, with *R. microplus* being the most commonly found species.

**Table-2:** Distributions number of tick species in the study area.

Spesies	Percentage of total ticks (%)
*R. pilans*	15/1575 (0, 95)
*H. bispinosa*	421/1575 (26, 73)
*R. microplus* (Boophilus)	1139/1575 (72, 32)

*R. microplus*=*Rhipicephalus microplus*, *R. pilans*=*Rhipicephalus pilans*, *H. bispinosa*=*Haemaphysalis bispinosa*

**Figure-2:**
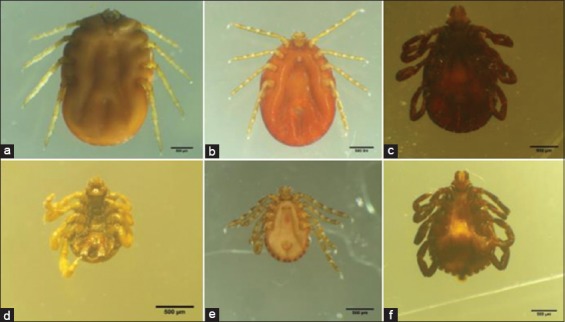
Representative pictures of identified different tick species of cattle during study in the area; (a) *Rhipicephalus* *microplus* ventral side of female, (b) *Haemaphysalis* bispinosa ventral side of female, (c) *Rhipicephalus pilans* ventral side of female, (d) *Rhipicephalus microplus* ventral side of male, (e) *Haemaphysalis bispinosa* ventral side of male, (f) *Rhipicephalus pilans* ventral side of male. bar 500 μm.

The two genera were differentiated by the presence of an ornate scutum. The *Rhipicephalus* genus exhibited an inornate scutum, a hexagonal basis capituli with sharp lateral angles, short anterior palps, eyes, a rounded and comma-shape of spiraclum, and festoons. Specific features of the *Haemaphysalis* genus included, among others, a short basis capituli, a second palpal segment, leading laterally, no eyes, an inornate scutum, and festoons. With regard to the species, *R*. *microplus* exhibited a hypostomal dentition 4/4, with no bristle-bearing protuberance on the internal margin of the first palpal segment in both male and female specimens. Coxa 1 spurs were distinct, and the posterior lip of the female genital aperture was U shaped (Figure-2a). The male specimens showed caudal appendages and the lack of distinct spurs at the end of the adanal plates (Figure-2d). *R. pilans* was identified on the basis of a hexagonal basis capituli and coarse punctations on the scutum. There were two long spurs on the ventral sides of coxae I. The male specimens exhibited sickle-shaped adanal plates with a very concave internal margin. Both male and female specimens exhibited a rough and irregular scutum with large and conspicuous hair (Figure-2c and f). *H. bispinosa* could be distinguished on the basis of the palpal segments. There were two broad segments (Figure-2b and e) with a rectangular basis capituli (straight lateral margins). Eyes were absent and festoons were present. Ventral plates were absent in the male specimens. The posteroventral spurs of the third palpal segment were broad, triangular, and blunt in both sexes. Furthermore, both sexes exhibited a hypostomal dentition 4/4, with a conspicuously long and pointed spur on coxa I and short, blunt, ridge-like spurs on coxae II to IV.

## Discussion

In the present study, we successfully identified three tick species in 26 local cattle from five regions in Indonesia. The study was descriptive because the infestation of hosts was not uniform and the sampling period was short. If the sampling period was longer, more species might have been identified. Nevertheless, our findings suggest that the distribution map for ticks that infest cattle in Indonesia has changed in comparison with that reported previously [8].

For several years, cattle have been playing an important role in farming in rural areas. Tenant farmers in Java generally possess two to four cattle, while those in other areas (Timor, including West Nusa Tenggara and East Nusa Tenggara) possess 5-50 cattle because they have wider lands. Extensive, intensive, and semi-intensive cattle raising systems can be seen in Indonesia.

In the extensive cattle raising system, cattle are set free and allowed to graze on naturally growing grass or plants that are not used for agriculture [12]. Mating, raising, and fattening are all organized on the grazing land. The advantage of this system is a very low production cost.

In the intensive cattle raising system, cattle are raised in special stalls. This system is found in various villages in Java. The semi-intensive cattle raising system refers to a mixed raising system, where farmers raise a number of cattle for fattening with the existing animal feed in or around the agricultural area.

Ticks are widely spread in the semi-extensive cattle raising system, with *R. microplus* being the most common and well-documented species. This species is found in Indonesia and India [13,14], and it is assumed to spread *Babesia bovis* and *Babesia bigemina* among cattle [15]. The former *Babesia* species commonly infect cattle in Indonesia. Serological analysis has shown that the prevalences of *B. bigemina, B. bovis*, and a combination of both are 27.5%, 69.8%, and 25.7%, respectively [16]. *H. bispinosa* has been reported in India, Sri Lanka, Myanmar, Pakistan, Nepal, Australia, and Indonesia [17]. At present, the incidence of *H. bispinosa* tick infestation in Indonesia, especially in the Java region, is very high. The species was found in Malaysia as a vector of *Bartonella bovis*, but its role in Indonesia is still unknown [18]. With regard to *R. pilans*, there is no information regarding its prevalence or status as a vector for *B. bovis* in Indonesia, although it has been reported in the Philippines and Indonesian islands [4,17]. Five hosts for *R. pilans* have been suggested; these include cattle, water buffaloes, horses, pigs, and dogs [19]. However, there is no information on the role of this species as a vector for various diseases, and there are no reports stating that it bites humans [20].

## Conclusion

Our findings revealed that cattle in different regions of Indonesia were infested by variable numbers of tick species. In particular, the cattle in Yogyakarta and Riau were solely infested by *H. bispinosa*; this is a new finding in terms of the distribution of tick species in the country. To summarize, tick species belonging to the *Rhipicephalus* and *Haemaphysalis* genera currently infest cattle in Indonesia, with the infestation level varying among regions. These findings can help in the formulation of an effective strategy for controlling and preventing cattle tick infestation in the country.

## Authors’ Contributions

The research was determined, managed, and supervised by DP. AS and DP took samples, recorded samples, and samples analysis. AS and GP identified and analyzed data. YRN and JP wrote the report. All authors have observed and ratified the final manuscript.
